# An exploration of the postural, location- and social contact- related sub-characteristics of inactive but awake behaviour as a depression-like indicator in mice

**DOI:** 10.1016/j.applanim.2024.106431

**Published:** 2024-12

**Authors:** Anna C. Trevarthen, Agustina Resasco, Emily M. Finnegan, Elizabeth S. Paul, Michael T. Mendl, Carole Fureix

**Affiliations:** a University of Bristol, Bristol Veterinary School, Langford House, Langford BS40 5DU, United Kingdom; b Biological Research Facility, The Francis Crick Institute, 1 Midland Rd, London NW1 1AT, United Kingdom

**Keywords:** Depression, Behaviour, Inactive but awake, Posture, Mouse

## Abstract

Inactive behaviour is essential to life. However, specific forms of inactivity may be indicative of compromised welfare in certain captive conditions. Inactive but awake behaviour (IBA - spontaneous, motionless awake behaviour without interacting with the surroundings) has been documented in some species and may be associated with poor welfare and negatively valenced affective states. In our previous work in laboratory mice, we have identified environmental risk factors (non-enriched housing) and curative factors (antidepressant drug Venlafaxine) for IBA and we hypothesise that greater levels of IBA may represent a depression-like state in this species. Here we aimed to identify which specific sub-characteristics of IBA would show construct validity as a depression-like state by exploring the posture (i.e. lying, curled lying or sitting), social contact position (i.e. in physical contact with a cage mate or not) and location of mice while performing the behaviour during two experiments (respectively investigating the aetiology and the curative factors of IBA). In both experiments we expected that more IBA would be displayed in standard (non-enriched) laboratory cages, compared with large highly-enriched cages and that a move from a highly-enriched to a non-enriched cage would increase IBA, while the opposite treatment would result in a decrease. In our second experiment (curative factors investigation), we predicted that less IBA would be displayed by mice that voluntarily ingested an antidepressant (Venlafaxine) *versus* a placebo. Because we could not control the number of instances of each IBA sub-characteristic we measured and we had no a *priori* predictions about which IBA sub-characteristics would match our general IBA treatment predictions, we compared the effect size and the direction of the effect between our treatment groups to explore which of the sub-characteristics matched our general IBA predictions. Overall, we found little variation in the location IBA was performed, with the majority being seen in the nest. Across treatment comparisons in both experiments, overall, the largest effect sizes were measured for IBA performed when in contact with the cage mate and performed when lying and both characteristics generally matched the direction of our treatment-related predictions. We suggest that future work should perform more detailed analyses of the specific characteristics of IBA by identifying behavioural sequences and the co-occurrence of the sub-characteristics to obtain a more complete picture of IBA as a depression-like indicator.

## 1 Introduction

Inactive behaviour is an important, restorative aspect of animal life, but inactivity has other functions and can take many forms (Fureix and Meagher, 2015). One specific form of inactivity has been documented in the home environment of certain captive animal species, with some animals displaying spontaneous, motionless (but awake) behaviour and no interaction with their surroundings (e.g. Fureix et al., 2012; Fureix et al., 2016; Meagher and Mason, 2012; Hurst et al., 1999; Harvey et al., 2019; Hintze et al., 2020). The affective states which are associated with this unusually elevated form of inactivity (hereafter “inactive but awake behaviour”: IBA) remain unknown, although initial evidence indicates them to be negatively valenced and associated with poor welfare (Fureix and Meagher, 2015). Indeed, housing-induced differences in IBA have been reported, with increased levels being observed in barren (non--preferred, compared to enriched) housing in a number of animal species including pigs (Bolhuis et al., 2005, 2006), rats (Hurst et al., 1999), cattle (Hintze et al., 2020), horses (Fureix et al., 2012, 2015), mink (Meagher and Mason, 2012; Meagher et al., 2013), mice (Fureix et al., 2016) and primates (Hennessy et al., 2014). This has led researchers to hypothesise that greater levels of this specific form of inactivity could represent a behavioural indicator of a boredom-like (Burn, 2017; Meagher, 2019) or a depression-like state (*e.g*. Fureix et al., 2015; MacLellan et al., 2021) in animals. The current paper relates to a programme of work testing the latter hypothesis (depression-like state) in mice.

The aetiology of IBA is starting to be researched and (as is found with human clinical depression – Sullivan et al., 2000; Saveanu and Nemer-off, 2012; American Psychiatric Publishing 2016) early-life stress, genetic susceptibility and/or chronic stress in later life are common triggers of the behaviour. For example, following post-natal separation from their mothers and subsequent rearing in barren wire cages, young rhesus monkeys were observed to be frequently inactive, displayed a fixed gaze and ‘stared into space’ (Harlow and Harlow, 1962). Similarly, spatially restricted laboratory rats show a form of IBA when group-housed in standard cages, with their eyes being open and stationary, but showing no directed attention (Hurst et al., 1999). So do mice housed in barren cages, even more so in some strains (hence suggesting genetic susceptibility) (Fureix et al., 2016; Nip et al., 2019, Trevarthen et al., 2019). Greater levels of IBA in animals also co-varies with some symptoms which are indicative of depression in humans (MacLellan et al., 2021). For instance, horses displaying greater levels of IBA show anhedonia (a loss of pleasure) (Fureix et al., 2015) and higher levels of IBA in mice are associated with greater immobility in the forced swim test (which can be reduced by antidepressant drugs) (Fureix et al., 2016; Nip et al., 2019), reduced preference for sucrose (Trevarthen et al. in prep), as well as two homeostatic symptoms of the human illness, *i.e*. sleep and weight changes (MacLellan and Mason, 2021). Additionally, our recent work, which demonstrated the alleviation of IBA using environmental enrichment and the antidepressant drug Venlafaxine in mice, provides further evidence for IBA as an indicator of a depression-like state (Fureix et al., 2022).

Because IBA shares features with other common inactive states, it is important that the species-specific characterisation of IBA is as detailed as possible to ensure it is not confused with other similar states (which may or may not share the same emotional valence). Some studies have reported postural changes during IBA for certain species. For example, horses exhibit a withdrawn posture (characterized by standing with eyes open, stretched neck (open jaw-neck angle) and similar height between the neck and back - Fureix et al., (2015)), which can be differentiated from other inactive postures (e.g. resting) (Fureix et al., 2012). Similarly in studies of rhesus macaques, having a hunched posture can be associated with ‘depressive-like’ inactive behaviour (Hennessy et al., 2014). Hintze et al., (2020) have gone one step further by observing head, ears and eyes and whether the animal was standing or lying). More recently, MacLellan et al. (2022) aimed to refine the IBA phenotype in mice, finding that IBA performed in a hunched posture consistently appeared to improve its accuracy as a depression-like indicator in Balb/c mice. Additionally, elevated levels of eye squinting during IBA was seen across three strains of conventionally housed mice (Balb/c, C57BL/6 and DBA/2). However, this result was not replicated across a second cohort of mice (MacLellan et al., 2022). Other than posture and facial expression, few studies have attempted to refine IBA measurement further by identifying the additional specific characteristics which accompany the behaviour (e.g. location in cage or contact with cage mate). Meagher et al., (2013) and Meagher et al., (2017) identified subtypes of inactivity by documenting both the posture and location of awake and sleeping mink and suggested that overall lying with the eyes open in the open cage (which was induced by reduced enrichment) was likely to be indicative of a boredom/depression-like state, whereas lying specifically in the nest-box was likely to be fear or anxiety-related.

Here, we aimed to explore in laboratory mice (*Mus musculus*) which, if any, sub-characteristics of IBA (namely the posture, physical contact with a cage mate, and the location in the cage) would show construct validity^1^ as indicators of a depression-like state. We re-used data which had been collected across two different experiments (one investigating the aetiology (Trevarthen et al. *in prep*) and one examining the curative factors of IBA (Fureix et al., 2022)). Investigations related to IBA behaviour and its relationship with depression-like conditions are relatively recent, and at this stage, we had no *a priori* predictions about which *specific* sub-characteristics might show construct validity of depressive-like IBA. However, we had predictions relating to whether IBA levels should be increased or decreased by risk and curative factors, respectively. Due to the exploratory nature of the experiment, we had no control over the number of instances of each sub-characteristic we measured and therefore could not perform specific hypothesis testing due to a lack of statistical power. Instead, we compared the effect sizes generated from the treatment comparisons within each of our experiments for each sub-characteristic of IBA to assess whether the strength and direction of the effect matched our general IBA treatment-related predictions (see detailed predictions in Method section).

## 2 Material and methods

Data analysed in the current paper were collected from two different experiments; experiment 1 ran from October 2018 to January 2019 and experiment 2 ran from April to July 2019. The aims and methods of each experiment are summarised below; the detailed methodologies for both experiments are published elsewhere (experiment 2 – Fureix et al., 2022) or provided in the supplementary material (experiment 1).

### 2.2 Summary of experiment 1 and experiment 2 (Table 1, further details published in supplementary material and Fureix et al. 2022)

The aim of experiment 1 was to investigate environmental and genetic aetiological factors in the development of inactive but awake behaviour in mice, through working with two strains of mice (female C57BL/6 and DBA/2 J, equally spread across treatments) and the manipulation of their home-cage environment. As such, after an initial 3-weeks housing period in either a standard relatively barren ‘shoebox’ (‘NE’) or a larger, highly enriched (‘EE’) cage (phase 1), half of the mice were moved into an alternative environment, while all other mice remained in their initial environment (see Table 1). Mice then remained in this environment for a further three-weeks (phase 2). We predicted that IBA behaviour would be displayed more in NE than in EE conditions, that when mice were moved from an EE to a NE environment IBA would increase, and decrease when moved from NE to EE. Due to in-consistencies in the level of IBA displayed by each strain in previous work (sometimes higher in C57s: Fureix et al., 2016, Nip et al., 2019, MacLellan et al., 2022, sometimes higher in DBAs: Trevarthen et al. *in prep*; No strain difference observed: Fureix et al., 2022), it was not possible for us to make firm strain predictions in the current study.

The aim of experiment 2 was to test whether potential curative factors (common and effective in the treatment of clinical depression in humans) were effective at alleviating IBA in mice. This involved the investigation of the effect of an environmental enrichment manipulation (as per experiment 1) and a pharmacological intervention using an antidepressant drug i.e. Venlafaxine Hydrochloride (serotonin and norepinephrine reuptake inhibitor, *Tocris, Bio-Techne Ltd*, UK) on the performance of IBA behaviour. We also tested the prediction that Venlafaxine would prevent the rise in inactive but awake behaviour shown to happen following environmental enrichment removal (Fureix et al., 2022). During the first two weeks post-arrival, the mice were housed in either EE or NE cages. Female mice from the C57BL/6 and the DBA/2 J strains were studied (equally spread across treatments). All the mice were trained to self-administer a condensed milk vehicle (0.1 ml). Following this training phase, the mice remained in their original housing condition (either EE or NE) for a further three-week period (phase 1) – see Table 1. During phase 1, either a Placebo or Venlafaxine (at a concentration of 10 mg/kg) was added to the condensed milk (half of the mice from each housing condition were randomly allocated to receive either the drug or the placebo), but no therapeutic effect of Venlafaxine was expected to happen at this stage. Indeed, antidepressants commonly show delayed onset of actions e.g. Mwebe, (2018); Frodl, (2017), and as expected placebo-treated and venlafaxine-treated did not significantly differ in their IBA levels during phase 1 for either EE or NE housing conditions (Fureix et al., 2022). On day 35 of the experiment, half of the mice housed in NE cages were either relocated to EE cages or left in NE cages, balancing for pharmacological treatments (see Table 1). The mice housed in EE cages (of which half were from the placebo and half from the Venlafaxine treatment) were all relocated to NE cages, where they remained for a further three-weeks (phase 2). We expected that IBA initially triggered by the NE condition would be alleviated both by increasing enrichment (i.e. moving to EE) and by the antidepressant Venlafaxine. When mice were initially housed in an EE environment and moved into an NE condition, we expected IBA to increase for the placebo group but for Venlafaxine to prevent such an increase in the group given the drug. Again, due to inconsistencies in the level of IBA displayed by each strain in our previous work, it was not possible for us to make firm strain predictions in the current study.

### 2.3 Behavioural scan sampling

The behaviour relevant to the hypotheses being tested in both experiments was being *inactive but awake*, IBA, defined as ‘mouse motionless, muzzle in sight and eyes open’. In experiment 1, a 15 s focal observation period was used to confirm IBA, which was based on previous work (Fureix et al., 2016; Harper et al., 2015). However, during the course of experiment 1, additional focal observations were made by an independent observer to document the length of each individual IBA bout. These observations revealed that a (less conservative) shorter duration of IBA was more appropriate and hence the 15 s focal observation was reduced to 3 s for experiment 2 (see Supplementary Material).

In the present paper, we explored the specific characteristics of IBA in terms of the location within the cage, the posture and whether the mouse was in contact with its cage-mate for each bout of the behaviour observed (see Table 2 for the specific characteristics ethogram used). Behaviour was recorded during the dark phase *via* live scan-sampling (Martin and Bateson 2007), switching from scan to focal sampling to allow for differentiation between behaviours characterised by a lack of movement (*e.g*. IBA *versus* sleeping) as in *e.g*. (Fureix et al., 2016; Harvey et al., 2020). Details of sampling methods and numbers of scans collected for each experiment are presented in Supplementary Material and Fureix et al., (2022).

### 2.4 Statistical analyses

**Fig. 1:**
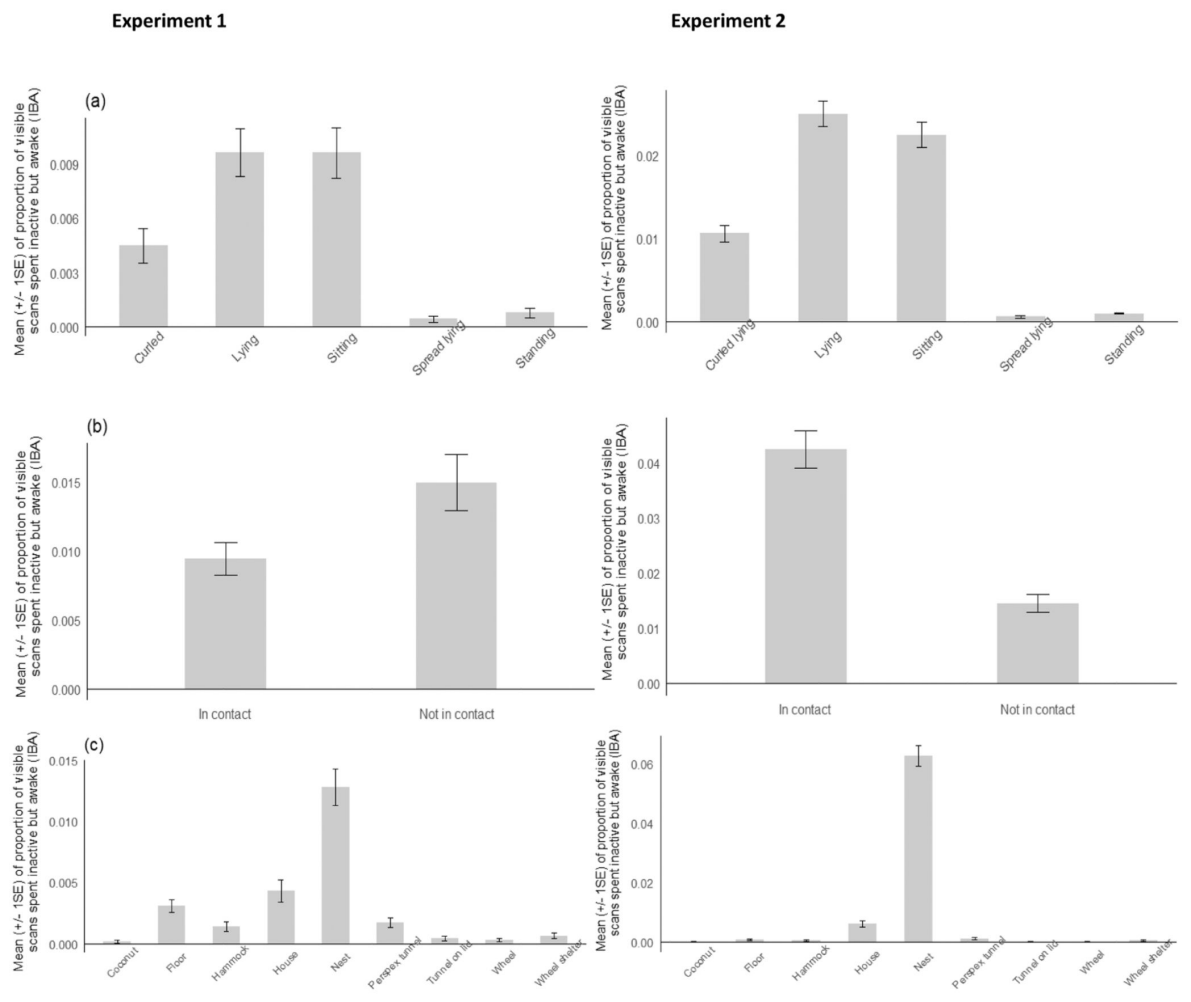
Mean (+/- 1SE) proportion of visible scans spent displaying IBA for (a) posture, (b) social contact and (c) location. Further analysis was not performed on standing and spread lying postures due to their relative infrequent display. The majority of IBA was performed in the nest, house or on the cage floor (all three locations were used for nesting; the cage floor was particularly used by NE mice for nesting in the absence of numerous options) and it was therefore concluded that there was little variation in the location IBA was performed.

During behavioural observations in experiment 1, we recorded 790 IBA bouts which were included in analyses (minimum IBA threshold of 15 s used). In experiment 2, we used a shorter duration criterion for our IBA definition (minimum IBA threshold of 3 s used) and hence recorded more bouts (1577) which were included in the analyses. These different thresholds prevent numerical comparisons (i.e. pooling data for analyses) between datasets. It was not always possible to record the social contact position because there were occasions when the cage-mate was not visible (for example hiding in the nest), although this happened rarely (exp 1: 18 times out of 790 bouts; exp 2: 68 times out of 1577 bouts). The postures “spread-lying” and “standing” were observed infrequently during both experiments and therefore were not analysed further (spread lying exp 1: 12 out of 790 bouts, exp 2: 17 out of 1577 bouts; standing exp 1: 17 out of 790, exp 2: 24 out of 1577). For both experiments, there was not enough variation in the location in which IBA was performed to carry out further analyses (most IBA was performed in the nest, Fig. 1c).

The proportion of visible scans spent performing IBA for each remaining sub-characteristic of posture and social contact (*i.e*. posture: lying, curled lying, sitting; social contact: in contact or alone) was calculated for each mouse during each phase (phase 1: pre and phase 2: post-environmental shift) for both experiments. Data from the two different strains of mice were pooled for these analyses because of lack of statistical power to investigate this factor. Indeed, the sub-characteristic analyses conducted here were exploratory in design, with no control over the number of instances mice of each strain displayed each IBA sub-characteristic, making it challenging to test the strain hypothesis.

Although we had general predictions for how each treatment might influence IBA performance for each experiment, we had no *a priori* predictions about which specific *sub-characteristics of* IBA in terms of posture, physical contact with cage mate and location in the cage might provide construct validity for IBA as a depression-like condition. We also had little control over the number of instances of each sub-characteristic we observed. For these reasons we did not conduct formal statistical analyses (hypothesis testing) on the sub-characteristic data and instead took an exploratory approach by calculating the effect size of the difference between our treatment groups of interest for each sub-characteristic.

We first calculated the mean difference in the proportion of visible scans spent performing IBA between the EE and NE groups for each specific sub-characteristic during phase 1 of each experiment. We then calculated the Cohen’s d effect size using the mean difference between our housing treatments and the respective pooled standard deviation. Finally, we used R Studio version 4.2.1 (Psych package) to calculate the confidence interval for the effect size comparison.

We made similar calculations to investigate the change in the mean proportion of visible scans spent performing IBA for each specific sub-characteristic between phases of each experiment (i.e. before and after manipulation), for each treatment group (see Table 1 for treatment group descriptions for each experiment). We similarly calculated the Cohen’s d effect size using the mean difference in the treatment groups of interest (we did not compare every treatment combination, only those for which we had specific directional *a priori* predictions about the *change* in IBA between phases of the experiment i.e. those which would provide information about the construct validity of each sub-type of IBA as an indicator of a depression-like state) and the pooled standard deviation for each sub-characteristic treatment comparison (5 treatment comparisons for experiment 1 and 6 treatment comparisons for experiment 2 – **see** Tables 6 and 7 **for comparisons and predictions**). We again used R Studio (version 4.2.1) to calculate the confidence interval for the effect size comparison. For each set of analyses, we adopted a commonly used framework as suggested by Cohen (1988) to give an approximate magnitude of the effect size strengths (strong effect: d≥0.8; medium effect: d≥0.5; small effect: d≥0.2). To allow for a simplified assessment of the strength of each sub-characteristic as a representative of depressive-like IBA, an overall mean Cohen’s d value was calculated for each sub-characteristic of IBA. To do this, we used the modulus of each Cohen’s d value (as only the strength of the effect size, rather than the direction was important for this assessment) and the mean d was calculated.

## 3 Results

While performing IBA, the mice varied in their curled, lying, and sitting postures (Fig. 1a), and in being or not being in contact with the cage-mate (Fig. 1b). Postural sub-characteristics were not independent from contact positions (Table 3). However, although lying was more often displayed in contact with a cage mate in both experiments, this association was in the opposite direction across experiments for the curled and sitting postures (both postures expressed more often not in contact than in contact in Experiment 1, whereas they were expressed more often in contact than not in contact in Experiment 2). As mentioned in the Methods, there was little variation in the location IBA was performed, with the majority being performed in the nest (Fig. 1c).

The effect sizes and confidence intervals for each sub-characteristic during phase 1 are presented in Table 4 (for experiment 1) and Table 5 (for experiment 2). When interpreting the results, it is important to note that wide confidence intervals were generated for many of the effects. This is likely due to the small sample sizes used in the effect size calculations, so large effect sizes should be interpreted with a higher degree of uncertainty. For experiment 1, the Cohen’s d value indicates that a strong effect was measured for IBA in contact with the cage mate and for lying IBA, whilst a medium effect was measured for sitting IBA and weak effects for curled IBA and IBA not in contact with the cage mate. For experiment 2, however, strong effect sizes were measured for IBA not in contact, lying, sitting and curled IBA, while a medium effect was recorded for IBA in contact with the cage mate. For both experiments the *direction* of the effects matched our treatment related predictions consistently (i.e. that greater levels of IBA would be recorded in the NE compared to the EE condition).

**Fig. 2:**
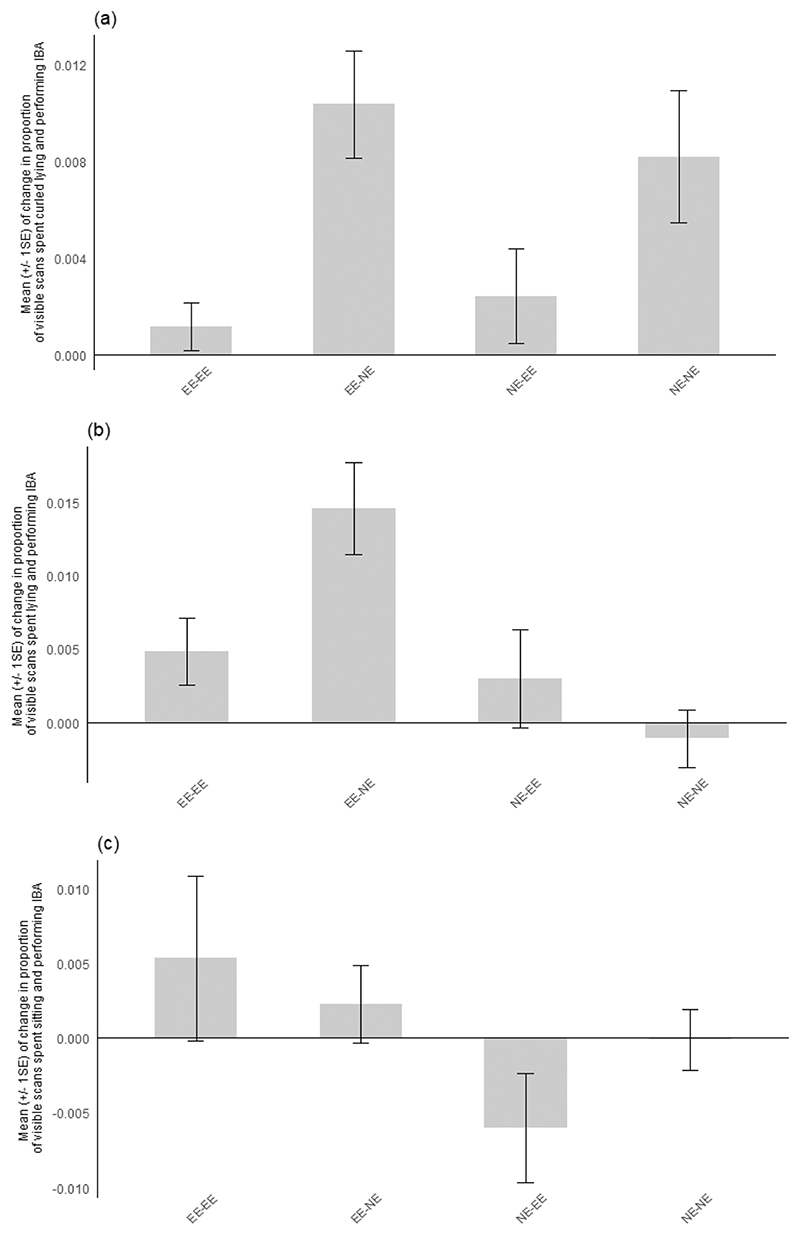
Mean (+/- 1 SE) of change between phases in the proportion of visible scans spent performing Inactive but Awake behaviour (IBA) with the associated sub-characteristic postures **a)** curled-lying, **b)** lying and **c)** sitting for each treatment group in experiment 1. **EE-NE**: mice initially housed in enriched cages and moved to non-enriched cages; **EE-EE**: mice housed in enriched cages throughout the experiment; **NE-NE**: mice housed in non-enriched cages throughout the experiment; **NE-EE**: mice initially housed in non-enriched cages and moved to enriched cages.

**Fig. 3:**
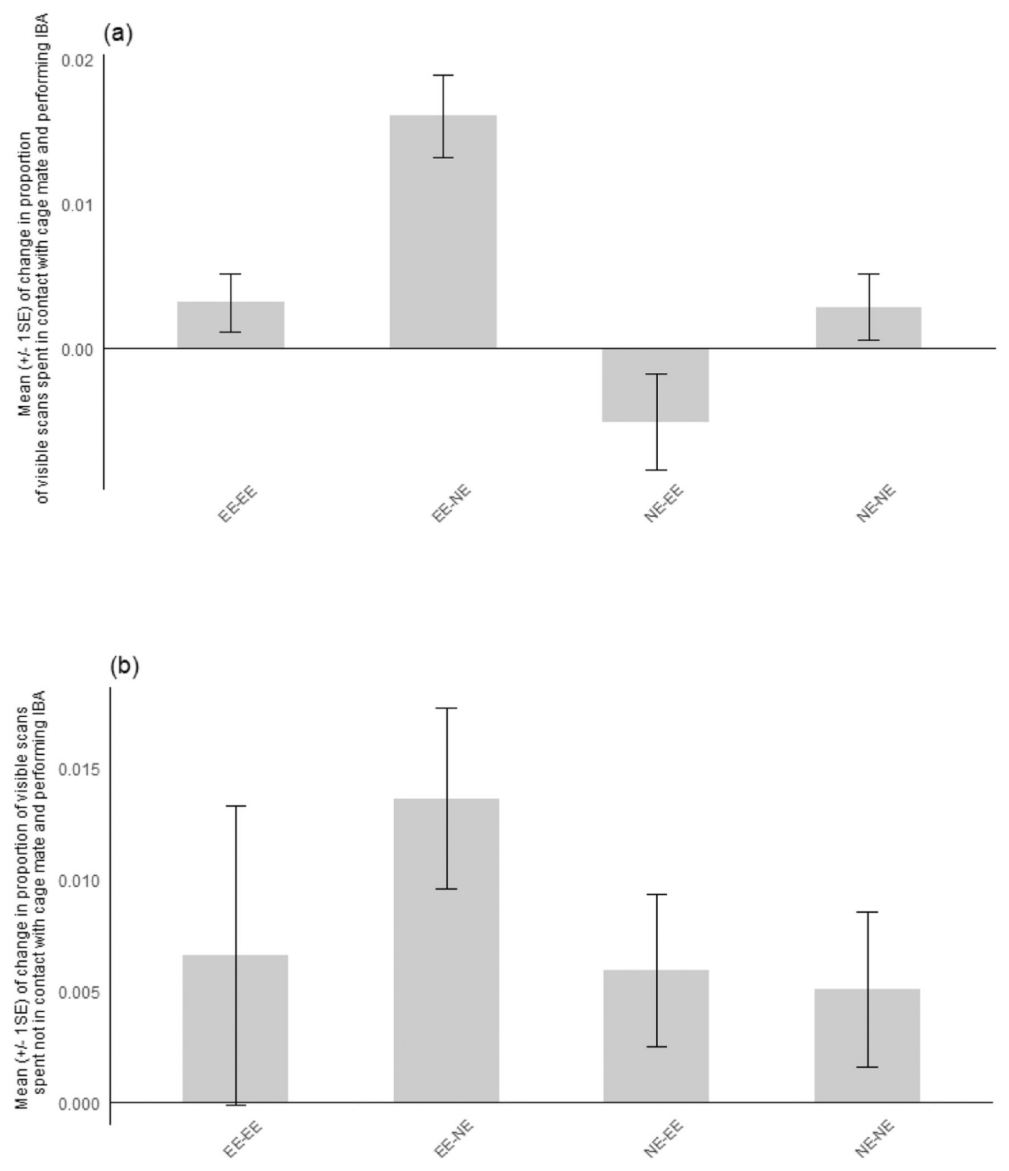
Mean (+/- 1 SE) of change between phases in the proportion of visible scans spent performing Inactive but Awake behaviour (IBA) with the associated sub-characteristic contact positions **a)** in contact and **b)** not in contact with the cage mate for each treatment group in experiment 1. **EE-NE**: mice initially housed in enriched cages and moved to non-enriched cages; **EE-EE**: mice housed in enriched cages throughout the experiment; **NE-NE**: mice housed in non-enriched cages throughout the experiment; **NE-EE**: mice initially housed in non-enriched cages and moved to enriched cages.

**Fig. 4:**
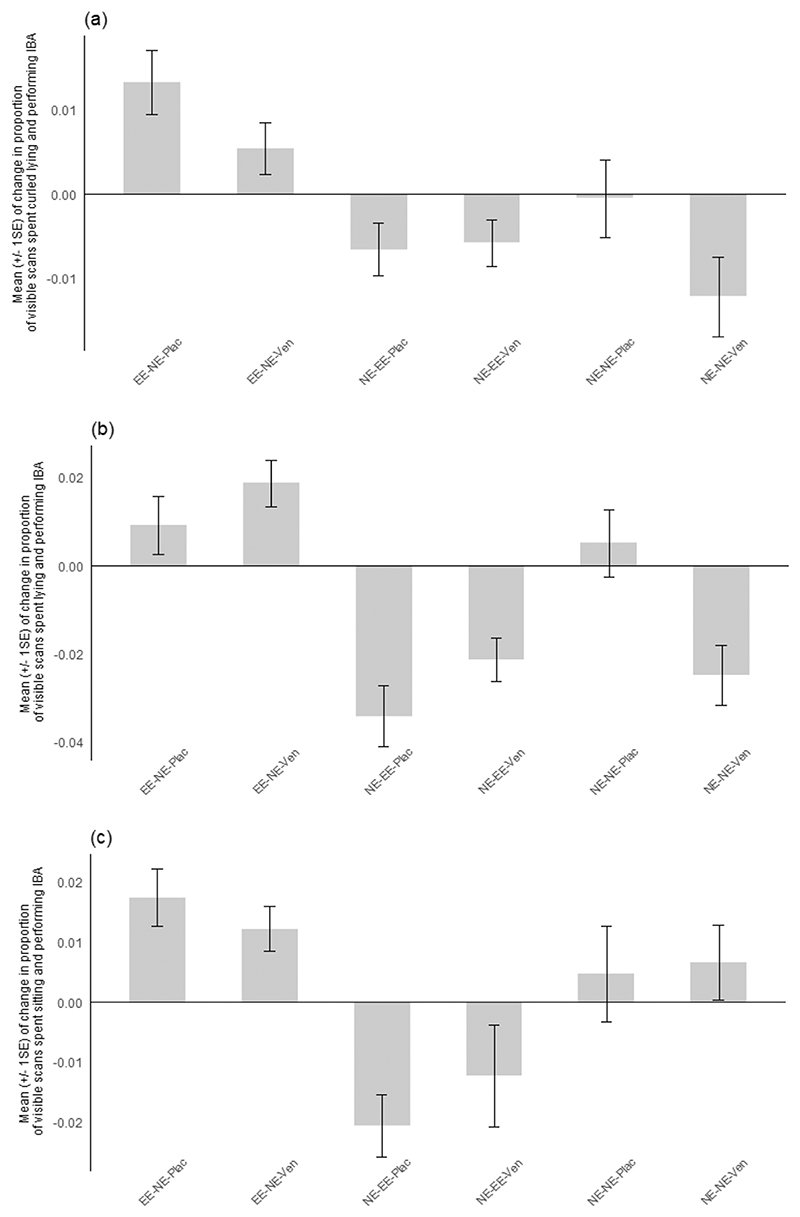
Mean (+/- 1 SE) of change between phases in the proportion of visible scans spent performing Inactive but Awake behaviour (IBA) with the associated sub-characteristic postures **a)** curled-lying, **b)** lying and **c)** sitting for each treatment group in experiment 2. **EE-NE-Plac**: mice initially housed in enriched cages and moved to non-enriched cages with placebo; **EE-NE-Ven**: mice initially housed in enriched cages and moved to non-enriched cages with Venlafaxine; **NE-EE-Plac**: mice initially housed in non-enriched cages and moved to enriched cages with placebo; **NE-EE-Ven**: mice initially housed in non-enriched cages and moved to enriched cages with Venlafaxine; **NE-NE-Plac**: mice housed in non-enriched cages throughout the experiment with placebo (control); **NE-NE-Ven**: mice housed in non-enriched cages throughout the experiment with Venlafaxine.

**Fig. 5:**
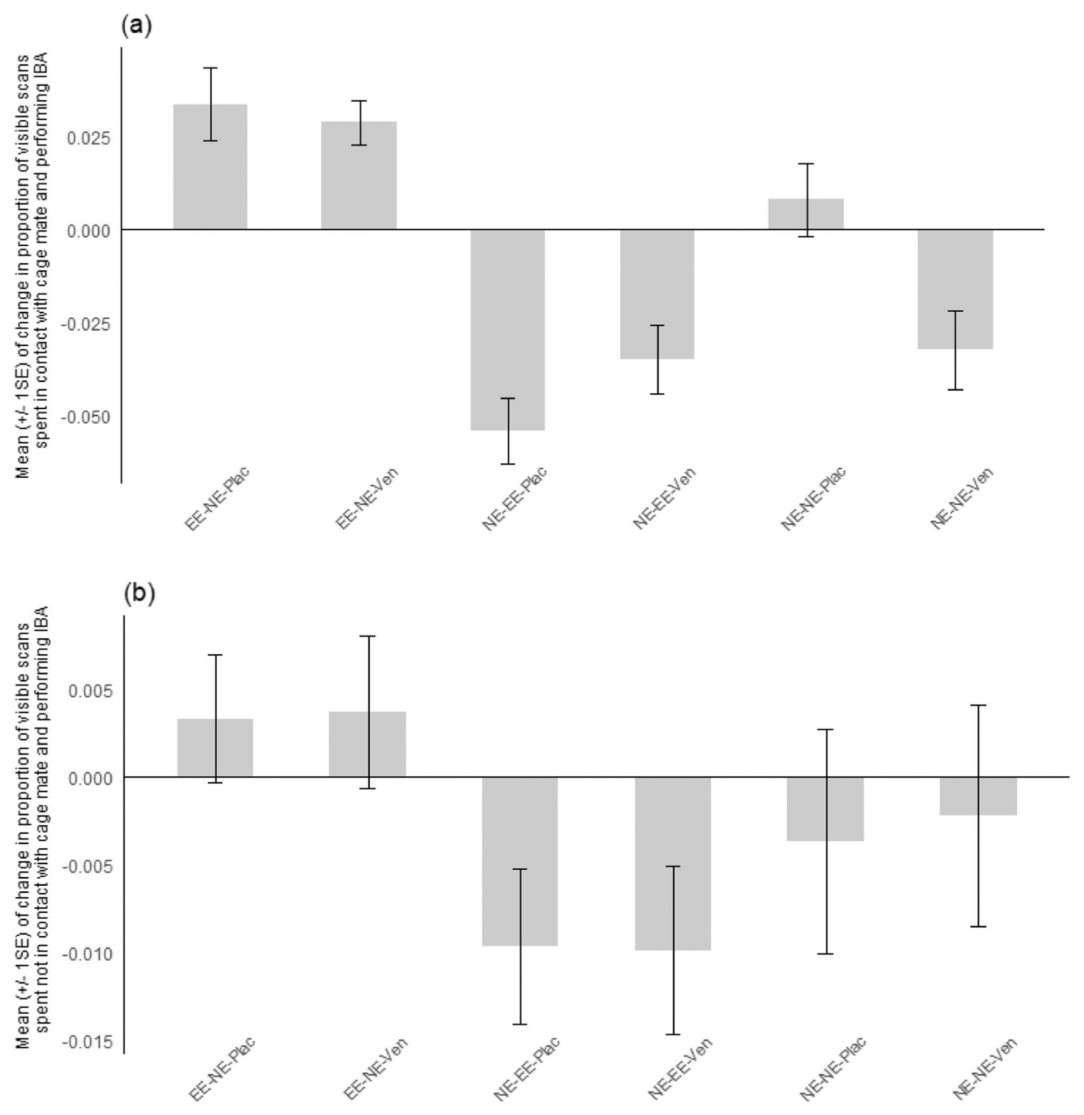
Mean (+/- 1 SE) of change between phases in the proportion of visible scans spent performing Inactive but Awake behaviour (IBA) with the associated sub-characteristic contact positions **a)** in contact and **b)** not in contact with the cage mate for each treatment group in experiment 2. **EE-NE-Plac**: mice initially housed in enriched cages and moved to non-enriched cages with placebo; **EE-NE-Ven**: mice initially housed in enriched cages and moved to non-enriched cages with Ven-lafaxine; **NE-EE-Plac**: mice initially housed in non-enriched cages and moved to enriched cages with placebo; **NE-EE-Ven**: mice initially housed in non-enriched cages and moved to enriched cages with Venlafaxine; **NE-NE-Plac**: mice housed in non-enriched cages throughout the experiment with placebo (control); **NE-NE-Ven**: mice housed in non-enriched cages throughout the experiment with Venlafaxine.

The mean change in IBA for each sub-characteristic is displayed across Figs. 2–5. Effect sizes and 95 % confidence intervals for the comparison between housing-related treatments between pre- and post-manipulation phases for each sub-characteristic of IBA are presented in Table 6 (experiment 1) **and** 7 (experiment 2). The magnitude of the overall mean Cohen’s d value for each sub-characteristic indicates that a strong effect was shown for IBA in contact with the cage mate across both experiments and that medium effect sizes were shown for lying and curled IBA in both experiments. Medium-weak effect sizes were displayed for sitting IBA, whilst a weak overall effect size was shown for IBA not in contact across treatment comparisons for both experiments.

In terms of the *direction* of the effects, green highlighted cells in

Tables 6 and 7 indicate a match with our general treatment-related IBA predictions (although this cannot be tested statistically here). Overall, most sub-characteristics across both experiments match with the direction of our treatment-related predictions if IBA represents a depression-like condition. Moreover, IBA in contact with the cage mate (which also exhibits the strongest effect sizes) was the only sub-characteristic to match the direction of our experimental predictions for all treatment comparisons and for both experiments. For experiment 2 only, curled IBA matched with the direction of all our treatment-related predictions.

## 4 Discussion

This study aimed to explore whether any sub-characteristics (posture, physical contact with a cage mate and location in the cage) of IBA have construct validity as indicators of a depression-like state in mice. We used data derived from two experiments, one exploring the aetiology of IBA and the other exploring its curative factors. Overall, we found that strong effect sizes were largely produced for IBA performed in contact with the cage mate when comparing treatments, across both experiments (although a medium effect was measured for the phase 1 treatment comparison for experiment 2). Lying IBA showed the strongest postural effect sizes across comparisons, while medium effects were mainly seen for curled IBA and weak effects were largely shown for sitting IBA. Similarly, in both experiments weak effect sizes were largely displayed for IBA not in contact with the cage mate (although during phase 1 of experiment 2 a strong effect was measured). The *direction* of the effect sizes largely matched our general IBA treatment-related predictions for both experiments, but particularly for IBA in contact with the cage mate, which universally matched our predictions across treatment comparisons and across experiments. The consistency of these results may provide a preliminary indication that the measure of IBA may be further refined using specific sub-characteristics of the behaviour (namely, being in contact with the cage mate and to a lesser extent, being in a lying posture).

However, these results are exploratory and several factors should be considered. Firstly, it is possible that the sub-characteristics tested here could have different behavioural functions. That the majority of IBA was conducted in either the nest or the house is interesting; Meagher et al., (2013) also found that mink commonly displayed IBA in the nest. However, it is possible that some of the IBA measured in these locations (particularly while lying or curled-lying) was in fact resting/pre- or post-sleeping behaviour (i.e. not related to a depression-like state), while the general predictions for these experiments were generated from work aiming to test IBA as a depression-like indicator (i.e. not related to resting). It has also been postulated by others that IBA could represent a boredom-like condition (Burn, 2017; Meagher et al., 2019) and future work is required to further differentiate indicators of boredom-like and depression-like states in mice. Weaker effect sizes were mostly shown for sitting IBA and it is possible that this behaviour is associated with some other aspect of behaviour, for example a transition between behaviours. To provide clarification, is it essential that in future work, behavioural transitions are studied by recording sequences of behaviour in the home cage.

Secondly, we monitored a limited range of sub-characteristics of IBA here and it may be that different (perhaps more subtle) sub-characteristics are more representative of IBA as a depression-like condition. A recent study of mice has found that hunched postures could be indicative of IBA as a depression-like condition in Balb/c mice, although this result was not consistently observed in C57BL/6 and DBA/2 strains (MacLellan et al., 2022). Similarly, recent work on rats suggests that a more subtle sub-type of IBA, namely with ocular squinting, may be more representative of a welfare indicator in that species (Young et al., 2024). These were both sub-characteristics which were not recorded in our current work, and it would be beneficial to investigate these recent findings further. Indeed, literature from non-rodent species provides strong evidence that postural (or other characteristic) changes occur in a state of poor welfare. For example, a slumped or hunched posture is a diagnostic feature of mild-moderate depression in humans (Wilkes et al., 2017; Canales et al., 2010) and research suggests that adopting a more upright posture can have a positive effect on fatigue, affect and focus in clinical patients (Wilkes et al., 2017). Similarly, gait can be affected by depression with patients walking more slowly, displaying reduced arm swinging, greater lateral body movements and a more slumped posture than non-depressed subjects (Feldman et al., 2020; Michalak et al., 2009). While these postures are not directly comparable to postures displayed by mice in the current experiments, the studies on humans do suggest that postural differences might also be displayed by animals in a depression-like state. Indeed, studies on negatively-valenced states in horses, cattle, mink and macaques have identified such postures. Horses which underwent chronic environmental stress displayed an atypical ‘withdrawn’ posture (Fureix et al., 2015) and individually housed macaques (a social species) exhibited a hunched posture (Hennessy et al., 2014, 2017). Interestingly, in studies aiming to detail specific postural features of bored or depressed-like conditions in mink and cattle, lying with open eyes was found to be most commonly displayed in the least enriched and most intensive housing conditions (Meagher et al., 2013; Hintze et al., 2020). These results match with the strongest effect sizes among postures we have explored in our current work, although clearly there are species-specific differences in the postures performed during IBA. Hence, future work should aim to expand on the postural ethogram further in mice.

Thirdly, it is important to consider that the postural and social sub-characteristics measured here are not mutually exclusive (i.e. mice performed lying IBA whilst in contact with the cage mate more often than not in contact). Moreover, we were also not able to document here which cage-mate initiated the contact and this information would be necessary to differentiate incidental contact from that apparently sought by the individual performing IBA. In order to disentangle these features, future work should aim to monitor full behavioural profiles, sequences of behaviour and the co-occurrence of sub-characteristics (e.g. Hintze et al., 2020).

Finally, the two studies presented here were exploratory in design and we had no control over the number of instances of each IBA sub-characteristic which was displayed. Due to the small sample size within this study, the effect sizes generated here should be interpreted with caution as there is an elevated risk of overestimation due to random variation. The effect sizes seen here are largely accompanied by wide confidence intervals, indicating increased uncertainty in the true effect size. Having increased control over the performance of IBA would increase the power of the study design and make statistical testing of the hypotheses possible. One approach to improve the experimental design would be to use repeated measures to test within-individual responses (i. e. animals experience each treatment condition). Additionally, improvements could be made to the design of experiment 2 by adding an EE-EE (stably-housed with enrichment) control group to disentangle the protective/curative property of enrichment. Future work should additionally focus on a full postural and social contact time-budget for mice using detailed focal observations of individuals in systematically controlled treatment groups. This additional control would make it possible to explore other potential influencing factors such as the strain of mouse, differences in which we were not able to test here (due to lack of power). Previous work has found inconsistencies between C57 and DBA strains when studying IBA (C57 displayed greater levels: Fureix et al., 2016, Nip et al., 2019; DBA displayed greater levels: Trevarthen et al., 2019; No strain difference observed: Fureix et al., 2022). This highlights the importance of recording time-budgets and behavioural sequences for commonly used laboratory strains of mice.

In summary, consistently strong effect sizes were produced for IBA performed while in contact with the cage mate and followed the direction of our general IBA predictions in both experiments. In contrast, weak effect sizes were largely produced for IBA not in social contact across both experiments (although we did record one strong effect). Of the postures, the strongest effect sizes were shown for IBA lying, whilst largely medium and weak effect sizes were displayed for curled and sitting IBA respectively, although all three postures mostly followed the direction of our general IBA experimental predictions. Future work should focus on expanding knowledge of behavioural transitions and sequences by recording focal observations of mice. The ethogram could be further refined by adding additional postures and detail (e.g. hunched posture, eye squinting). This would allow for a more complete assessment of whether any sub-characteristics of IBA might indicate a depression-like condition in mice.

## Supplementary Material

Supplementary data associated with this article can be found in the online version at doi:10.1016/j.applanim.2024.106431.

Supplementary material 1

Supplementary material 2

## Figures and Tables

**Table 1:** Summary of number of treatment groups, sample sizes, and main predictions for experiment 1 and experiment 2. **NE**: relatively barren, ‘shoebox’ cage; **EE**: larger, highly enriched cage; **IBA**: Inactive but Awake behaviour, **Plac**: placebo, **Ven**: Venlafaxine.

	Experiment 1								
	Group	NE-NE	EE-EE		EE-NE		NE-EE		
	Group description	Mice housed in NE cages throughout	Mice housed in EE cages throughout		Mice initially housed in EE cages relocated to NE cages		Mice initially housed in NE cages relocated to EE cages		
	Group size	n = 16 mice	n = 14 mice		n = 16 mice		n = 16 mice		
	Main experimental predictions	→ NE cages would trigger greater time spent IBA compared to EE cages prior to environmental adjustment (phase 1 data only) → Time spent IBA would increase with environmental enrichment removal → Time spent IBA would decrease when providing environmental enrichment to mice housed in NE cages							
	**Experiment 2**								
	Group	**NE-NE-Plac**	**NE-NE-Ven**	**NE-EE-Plac**	**NE-EE-Ven**	**EE-NE-** **Plac**		**EE-NE-Ven**	
	Group description	Mice housed in NE cages throughout and given placebo	Mice housed in NE cages throughout and given Venlafaxine	Mice initially housed in NE cages relocated to EE cages and given placebo	Mice initially housed in NE cages relocated to EE cages and given Venlafaxine	Mice initially housed in EE cages relocated to NE cages and given placebo		Mice initially housed in EE cages relocated to NE cages and given Venlafaxine	
	Group size	n = 24 mice	n = 24 mice	n = 24 mice	n = 24 mice	n = 24 mice		n = 24 mice	
	Main experimental predictions	→ NE cages would trigger greater time spent IBA compared to EE cages prior to environmental adjustment (phase 1 data only)							
		→ Time spent IBA would decrease when providing environmental enrichment to mice housed in NE cages							
		→ Time spent IBA would decrease when administrating Venlafaxine							
		→ Venlafaxine would prevent the rise in IBA triggered by environmental enrichment removal							

**Table 2:** Ethogram used to identify the specific sub-characteristics of Inactive but awake (IBA) behaviour.

Characteristic	Sub-characteristic	Description
Posture	Sitting	Sitting with all four paws on floor, back arched
	Standing	Rearing on to back two paws. Front two paws touching an object, or free-hanging
	Lying	Lying straight with abdomen on floor
	Curled-lying	Lying with abdomen on floor in a ‘c’ shape with rear or front legs tucked to the side
	Spread-lying	Lying with abdomen on floor and with both rear and font legs extended outwards
Social contact	In contact	Physical contact with the cage-mate
	Not in contact	No physical contact with the cage-mate (i.e. distance between them)
Location	Nest	Mouse is in or on a piled-up area of
(* indicates enrichment locations which are specific to EE)		bedding/nesting substrate which has been moved into position by the mice.
	Floor	Mouse is on any part of the cage floor which does not contain an object or nest
	Perspex tunnel	Mouse is in or on top of the Perspex tunnel
	Cage lid	Mouse is suspended from the cage lid by gripping wire with paws
	Tunnel on lid*	Mouse is within the plastic transparent tunnel which is attached to the cage lid
	Hammock*	Mouse is within the suspended hammock
	Coconut*	Mouse is within the suspended half-coconut shell
	Wheel shelter*	Mouse is within the red shelter beneath the running wheel
	Wheel*	Mouse is on top of the running wheel
	House*	Mouse is in the cardboard house
	Other	Mouse is in a location not defined

**Table 3:** Contingency table displaying the variation in curled-lying, lying and sitting postures while in contact and not in contact with the cage mate in experiment 1 and 2. Observed frequencies in bold highlights whether being in social contact, or not, is the most frequently associated with each posture.

Experiment 1	${\text{chi}}_{2}^{2}$=68.3726, p < 0.00001			
	In contact	Not in contact	Goodness of fit ${\text{chi}}_{1}^{2}$	P
Curled-lying	53	**92**	10.49	0.0012
Lying	**170**	129	5.622	0.01774
Sitting	70	**224**	80.667	<0.00001
**Experiment 2**	${\text{chi}}_{2}^{2}$=72.9732, p < 0.00001			
	In contact	Not in contact	Goodness of fit ${\text{chi}}_{1}^{2}$	P
Curled-lying	**190**	92	34.057	< 0.00001
Lying	**555**	83	349.191	< 0.00001
Sitting	**373**	175	71.54	< 0.00001

**Table 4:** Effect size (Cohen’s *d*) and 95 % confidence intervals (upper and lower boundaries) for the comparison between housing-related treatments during **phase 1** of experiment 1 for each posture and contact sub-characteristics of IBA. *d* represents the mean difference in the proportion of visible scans each sub-characteristic (while performing IBA) was performed divided by the pooled standard deviation for the treatment comparison. Values highlighted in dark blue text indicate a strong effect size (≥0.8–1d.p) and values in light blue text indicate a medium effect size (≥0.5–1 d.p), whilst values which are ≥0.2 indicate a weak effect size. Cells highlighted in green are effect size comparisons for which the direction of the effect match our general IBA related predictions.

*Experiment 1*	Treatment comparison			
	**NE vs. EE**			
***Prediction***	**NE greater**			
***Sub-characteristic***	***Mean difference***	***d***	***CI lower***	***CI upper***
***Curled***	0.0013	0.40	-0.11	0.90
***Lying***	0.0065	**0.84**	0.32	1.36
***Sitting***	0.0039	0.46	-0.05	0.96
***In Contact***	0.0084	**1.25**	0.70	1.79
***Not in contact***	0.0035	0.35	-0.15	0.85

**Table 5:** Effect size (Cohen’s *d*) and 95 % confidence intervals (upper and lower boundaries) for the comparison between housing-related treatments during **phase 1** of experiment 2 (i.e. during initial feeding of Venlafaxine, but before the drug took effect – see Fureix et al. 2022) for each posture and contact sub-characteristics of IBA (Inactive but Awake). *d* represents the mean difference (pooled across drug treatments) in the proportion of visible scans each sub-characteristic (while performing IBA) was performed divided by the pooled standard deviation for the treatment comparison. Values highlighted in dark blue text indicate a strong effect size (≥0.8–1d. p) and values in light blue text indicate a medium effect size (≥0.5–1 d.p), whilst values which are ≥0.2 indicate a weak effect size. Cells highlighted in green are effect size comparisons for which the direction of the effect match our general IBA related predictions.

*Experiment 2*	Treatment comparison			
	**NE vs. EE**			
***Prediction***	**NE greater**			
***Sub-characteristic***	***Mean difference***	***d***	***CI lower***	***CI upper***
***Curled***	0.013	**0.81**	0.45	1.17
***Lying***	0.030	**1.13**	0.75	1.50
***Sitting***	0.023	**0.99**	0.63	1.36
***In Contact***	0.013	0.63	0.28	0.98
***Not in contact***	0.053	**1.00**	0.63	1.37

**Table 6:** Effect sizes (Cohen’s *d*) and 95 % confidence intervals (upper and lower boundaries) for the comparison between housing-related treatments in experiment 1 for each posture and contact sub-characteristic of IBA. *d* represents the mean difference in the **change** in each sub-characteristic (while performing IBA) between phases of the experiment (pre- and post-manipulation) divided by the pooled standard deviation for each treatment comparison. The mean Cohen’s *d* magnitude (ignoring the *direction* of the effect and focusing only on the strength i.e. taking the modulus) is also presented. Values highlighted in dark blue text indicate a strong effect size (≥0.8–1d.p) and values in light blue text indicate a medium effect size (≥0.5–1 d.p), whilst values which are ≥0.2 indicate a weak effect size. Cells highlighted in green are effect size comparisons for which the direction of the effect match our general IBA related predictions, whilst those highlighted in orange are those which do not match our predictions. We have not displayed treatment comparisons for which we could not make an *a priori* prediction about the direction of the effect.

*Experiment 1*	Treatment comparison																				
	**EE-NE vs. EE-EE**				**EE-EE vs. NE-EE**				**EE-NE vs. NE-EE**				**EE-NE vs. NE-NE**				**NE-NE vs. NE-EE**				
***Prediction***	**EE-NE greater change**				**EE-EE greater change**				**EE-NE greater change**				**EE-NE greater change**				**NE-NE greater change**				
***Sub-characteristic***	***Mean diff***	***d***	***CI lower***	***CI upper***	***Mean diff***	*d*	***CI lower***	***CI uppee***	***Mean diff***	*d*	***CI lower***	***CI upper***	***Mean diff***	***d***	***CI lower***	***CI upper***	***Mean diff***	***d***	***CI lower***	***CI upper***	**Mean Cohen’s magnitude**
***Curled***	0.009	1.32	0.51	2.10	-0.001	-0.20	-0.92	0.52	0.008	0.95	0.21	1.68	0.002	0.22	-0.48	0.91	0.006	0.61	-0.11	1.31	**0.66**
***Lying***	0.010	0.89	0.13	1.64	0.002	0.16	-0.56	0.88	0.012	0.89	0.16	1.61	0.016	1.49	0.69	2.27	-0.004	-0.38	-1.07	0.33	**0.76**
***Sitting***	-0.003	-0.19	-0.91	0.53	0.011	0.64	-0.10	1.38	0.008	0.65	-0.07	1.36	0.002	0.25	-0.45	0.94	0.006	0.50	-0.21	1.20	**0.45**
***In Contact***	0.013	1.31	0.51	2.09	0.008	0.75	-0.001	1.49	0.021	1.71	0.88	2.51	0.013	1.28	0.50	2.03	0.008	0.70	-0.02	1.41	**1.15**
***Not in contact***	0.007	0.34	-0.39	1.06	0.001	0.03	-0.68	0.75	0.008	0.51	-0.20	1.21	0.009	0.56	-0.15	1.27	-0.001	-0.061	-0.75	0.63	**0.30**

**Table 7:** Effect sizes (Cohen’s *d*) and 95 % confidence intervals (upper and lower boundaries) for relevant comparisons between treatment groups in experiment 2 for each posture and contact sub-characteristic of IBA. *d* represents the mean difference in the **change** in each sub-characteristic (while performing IBA) between phases of the experiment (pre- and post-manipulation) divided by the pooled standard deviation for each treatment comparison. The mean Cohen’s *d* magnitude (ignoring the *direction* of the effect and focusing only on the strength i.e. taking the modulus) is also presented. Values highlighted in dark blue text indicate a strong effect size (≥0.8–1d.p) and values in light blue text indicate a medium effect size (≥0.5–1 d.p), whilst values which are ≥0.2 indicate a weak effect size. Cells highlighted in green are effect size comparisons for which the *direction* of the effect match our general IBA related predictions, whilst those highlighted in orange are those which do not match our predictions. We have not displayed treatment comparisons for which we could not make an *a priori* prediction about the direction of the effect.

*Experiment 2*	Treatment comparison																								
	**EE-NE-Plac vs. EE-NE-Ven**				**EE-NE-Plac vs. NE-EE-Plac**				**EE-NE-Plac vs. NE-NE-Plac**				**EE-NE-Ven vs. NE-EE-Ven**				**NE-NE-Plac vs. NE-EE-Plac**				**NE-NE-Plac vs. NE-NE-Ven**				
***Prediction***	**EE-NE-Plac greater change**				**EE-NE-Plac greater change**				**EE-NE-Plac greater change**				**EE-NE-Ven greater change**				**NE-NE-Plac greater change**				**NE-NE-Plac greater change**				
***Sub-characterisiic***	***Mean diff***	***d***	***Cl lower***	***Cl upper***	***Mean diff***	**d**	***Cl lower***	**Cl upper**	**Mean diff**	**d**	**Cl lower**	**Cl upper**	**Mean diff**	**d**	**Cl lower**	**Cl upper**	***Mean diff***	**d**	***Cl lower***	***Cl upper***	**Mean diff**	**d**	***Cl lower***	***Cl upper***	**Mean Cohen’s *d* magnitude**
***Curled***	0.008	0.46	-0.11	1.03	0.020	1.16	0.54	1.77	0.014	0.66	0.08	1.24	0.011	0.78	0.19	1.36	0.006	0.31	-0.26	0.88	0.012	0.51	-0.07	1.08	**0.65**
***Lying***	-0.010	-0.33	-0.90	0.24	0.043	1.31	0.68	1.93	0.004	0.11	-0.45	0.68	0.040	1.61	0.95	2.26	0.039	1.11	0.49	1.71	0.030	0.85	0.25	1.43	**0.89**
***Sitting***	0.005	0.25	-0.32	0.82	0.038	1.56	0.90	2.20	0.013	0.40	-0.18	0.96	0.024	0.76	0.17	1.34	0.025	0.77	0.18	1.35	-0.002	-0.05	-0.62	0.51	**0.63**
**In contact**	0.005	0.12	-0.44	0.69	0.088	1.91	1.22	2.59	0.026	0.54	-0.04	1.11	0.064	1.69	1.02	2.34	0.062	1.37	0.73	1.99	0.040	0.82	0.22	1.40	**1.07**
**Not in contact**	- 0.0004	-0.02	-0.59	0.55	0.013	0.65	0.07	1.23	0.007	0.27	-0.30	0.84	0.014	0.61	0.025	1.18	0.006	0.22	-0.35	0.79	-0.002	-0.05	-0.61	0.52	**0.30**

## Footnotes

1 *Construct validity:* the degree to which animal-based measures adequately assess the construct of depression in this case (Mason and Mendl, 2025)
