# Supplementary material for: An exploration of the postural, location- and social contact- related sub-characteristics of inactive but awake behaviour as a depression-like indicator in mice
Source: Appl Anim Behav Sci. Author manuscript; Available in PMC 2025 Oct 22. (PMC7618284; doi:10.1016/j.applanim.2024.106431)
Supplement: Supplementary material 1 [file EMS207934-supplement-Supplementary_material_1.docx]

**An exploration of the postural, location- and social contact- related sub-characteristics of inactive but awake behaviour as a depression-like indicator in mice**

Anna C Trevarthen^*a^, Agustina Resasco^b^, Emily M Finnegan^a^, Elizabeth S Paul^a^, Michael T Mendl^a^, Carole Fureix^a^

^a^ University of Bristol, Bristol Veterinary School, Langford House, Langford BS40 5DU, United Kingdom

^b^ Biological Research Facility, The Francis Crick Institute, 1 Midland Rd, London NW1 1AT, United Kingdom

Authors’ email addresses: [anna.trevarthen@bristol.ac.uk](mailto:anna.trevarthen@bristol.ac.uk); [agustina](mailto:agustinaresasco@gmail.com).resasco@crick.ac.uk; emf4@hotmail.co.uk; [e.paul@bristol.ac.uk](mailto:e.paul@bristol.ac.uk); [mike.mendl@bristol.ac.uk](mailto:mike.mendl@bristol.ac.uk); [carole.fureix@bristol.ac.uk](mailto:carole.fureix@bristol.ac.uk).

* correspondence: Bristol Veterinary School, Dolberry Building, Langford House, Bristol, BS40 5DU, United Kingdom

**Supplementary material – Experiment 1 detailed methods**

1. Experiment 1 - Detailed Methods
   1. *Animals, housing, and husbandry*

Thirty-one female C57BL/6J (C57s hereafter) and 31 female DBA/2J (DBAs hereafter) mice (*Mus musculus*; Charles River, France; SPF) were pair-housed post-weaning in either highly enriched (N = 15 cages) or non-enriched (N = 16 cages) transparent open-top *Techniplast* cages. Estimates of animal numbers were based on power analyses using data from (Fureix *et al.*, 2016) (power 80%, significance criterion 2-tailed 0.05, Cohen’s d effect size 0.7 (minimum expected means difference 0.011 and estimated standard deviation 0.01562), replicated using several calculators). Upon arrival at the laboratory (at three-four weeks of age), each mouse was weighed and one C57 (black) and one DBA (brown) mouse of similar weight (+/- 2.5g) was pseudo-randomly allocated to each cage (mouse selected at random from mice of similar weights and allocated to the cage with a weight-matched cage-mate). The mixed-strain housing enabled individual identification within pairs, removing the need for invasive marking procedures (Walker *et al.*, 2013). The enriched cages (‘EE’ hereafter) were 44cm L x 34cm W x 20cm H and contained sawdust (*IPS*), two handfuls of nesting material, a plastic transparent shelter (∅ 15cm, 5.5cm H, *Biopac UK*), a red igloo with a running wheel (fast-trac, *Datesand*), a transparent polycarbonate handling tunnel (13cm L, ∅ 5cm, *Datesand*), a flexible transparent plastic tunnel (∅ 6cm, 30cm L) which was attached to the cage lid, three aspen gnawing blocks (two small: 5cm L x 1cm W x 1cm H, one large: 10cm L x 2cm W x 2cm H, *Datesand*), a small pine cone, two nestlets (*Ancare*, USA), a hammock (roughly 12 cm x 12 cm) made from a cotton sock attached to the lid, a sisal rope ladder and one half of a coconut shell (approximate dimensions: 12.7cm L x 7.6cm W x 5cm H, *Little Cherry Ltd*) which was attached to the lid with sisal rope. A millet spray (*Pets at Home*) was attached to the lid of each cage and was refreshed during monthly cage cleaning. Non-enriched cages (‘NE’ hereafter) were 37cm L x 21cm W x 14cm H and contained sawdust (*IPS*), one handful of nesting material, a small piece of cardboard (≈ 5 cm x 5 cm) for gnawing and a transparent polycarbonate handling tunnel (13cm L, ∅ 5cm, *Datesand*). Food (*LabDiet*) and water were available *ad-libitum* and animals were kept under a 12hr reversed light-dark cycle (lights on 1900-0700). All cages were housed within three scantainers (*Scanbur BK*) in a pseudo-random order that ensured an equal spreading of experimental treatments between the three scantainers, and within shelves for each scantainer. NE cages were cleaned every week and EE cages were cleaned every four weeks. Temperature (Mean ± SEM, 20.9± 0.5°C) and relative humidity were controlled at the room level (scantainers used as shelving units with doors open) and were checked daily.

Following three weeks of housing in their initial environment, half of the mice (8 EE and 8 NE cages) were moved into an alternative environment (*i.e.* NE if initially housed in EE, ‘EE-NE’; and EE if initially housed in NE, ‘NE-EE’), whilst all other mice stayed in their initial environment (7 control ‘EE-EE’ and 8 control ‘NE-NE’ cages). All mice were transferred to a clean cage regardless of whether their environment was being altered. Mice then remained in this environment for 8 weeks. When being moved between environments (and if handled during the experimental period) all NE mice were handled by the tail, whilst EE mice were handled using their home-cage Perspex handling tunnel, following a validated method shown to reduce stress in laboratory mice (Hurst and West, 2010; Gouveia and Hurst, 2013; Gouveia and Hurst, 2017).

- 1. *Behavioural scan sampling*

The behaviour relevant to the hypothesis under test was being *inactive but awake* (IBA hereafter), defined after Fureix *et al.* (2016), Harper *et al.* (2015) as ‘mouse motionless, muzzle in sight and eyes open, for at least 15s’. Behavioural observations of mice in their home cage began on day 4 after arrival, following three days of acclimatisation. All observations were conducted during the dark (active) phase under red ambient light, for three weeks, four days per week, over four 90-minute time blocks per day: 9:30-11:00, 11:30-13:00, 13:45-15:15, 15:45-17:15. Behaviour was recorded *via* live scan-sampling (Martin and Bateson, 2007), switching from scan to 15s focal sampling to allow for differentiation between behaviours characterised by a lack of movement (*e.g.* IBA *versus* sleeping) as in *e.g.* Fureix *et al.* (2016), Harvey *et al.* (2019). In total, 24 scan samples were taken per mouse each day, spread evenly across the four blocks (totalling 288 scan samples/mouse for this first phase of observation). During the first week of observations, two experimenters (AT, EF) were present during each scan to simultaneously record the behaviour of each mouse. Because inter-observer reliability analyses indicated a high degree of agreement (Cohen’s Kappa value: 0.978), all subsequent scan samples were split between two experimenters.

Environmental adjustment, *i.e.* moving into the alternative environment, or staying in the initial environment for control groups, happened on the day following the last day of observation, with a naïve experimenter pseudo-randomly switching the position of cages within each scantainer to ensure the behavioural observers were blind to the environmental adjustment each cage had undergone (treatments remained equally spread between the three scantainers and within shelves for each scantainer). Home-cage behaviour was monitored again for three consecutive weeks starting 2 days after environmental adjustment, following the above-described procedure (with the exception that scan samples were immediately split between two experimenters) and also totalling 288 scan samples/mouse during this second phase of observation.

**Supplemental Material section references**

Frodl, T. (2017) 'Recent advances in predicting responses to antidepressant treatment [version 1; peer review: 2 approved]', *F1000Res,* 6, pp. 619.

Fureix, C., Walker, M., Harper, L., Reynolds, K., Saldivia-Woo, A. and Mason, G. (2016) 'Stereotypic behaviour in standard non-enriched cages is an alternative to depression-like responses in C57BL/6 mice', *Behavioral Brain Research,* 305, pp. 186-190.

Gouveia, K. and Hurst, J. L. (2013) 'Reducing mouse anxiety during handling: effect of experience with handling tunnels', *PloS one,* 8(6), pp. e66401.

Gouveia, K. and Hurst, J. L. (2017) 'Optimising reliability of mouse performance in behavioural testing: the major role of non-aversive handling', *Scientific reports,* 7, pp. 44999.

Harper, L., Choleris, E., Ervin, K., Fureix, C., Reynolds, K., Walker, M. and Mason, G. (2015) 'Stereotypic mice are aggressed by their cage-mates, and tend to be poor demonstrators in social learning tasks', *Animal Welfare,* 24, pp. 463-473.

Harvey, N., D., Moesta, A., Kappel, S., Wongsaengchan, C., Harris, H., Craigon, P. and Fureix, C. (2019) 'Could greater time spent displaying waking inactivity in the home environment be a marker for a depression-like state in the domestic dog?', *Animals,* 9(7), pp. E420.

Hinchcliffe, J. K., Stuart, S. A., Mendl, M. and Robinson, E. S. J. (2017) 'Further validation of the affective bias test for predicting antidepressant and pro-depressant risk: effects of pharmacological and social manipulations in male and female rats', *Psychopharmacology,* 234(20), pp. 3105-3116.

Hurst, J. L. and West, R. S. (2010) 'Taming anxiety in mice by non-aversive handling', *Nature Methods,* 7(10), pp. 825-826.

Martin, P. and Bateson, P. (2007) *Measuring behaviour: an introductory guide. Third edition.* Cambridge: Cambridge University Press.

Mwebe, H. (2018) *Psychopharmacology: A mental health professionals guide to commonly used medications.* ST ALBANS: ST ALBANS: Critical Publishing.

Nip, E., Adcock, A., Nazal, B., Maclellan, A., Niel, L., Choleris, E., Levison, L. and Mason, G. (2019) 'Why are enriched mice nice? Investigating how environmental enrichment reduces agonism in female C57BL/6, DBA/2, and BALB/c mice', *Applied Animal Behaviour Science,* 217, pp. 73-82.

Trevarthen, A., Mendl, M., Finnegan, E., Paul, E., Resasco, A. and Fureix, C. (2019) 'Inactive but awake behaviour as a potential indicator of a housing-induced depressive-like state in mice'. *Universities Federation for Animal Welfare International Symposium 2019*, Bruges (Belgium), July 3rd – 4th.

Walker, M., Fureix, C., Palme, R. and Mason, G. (2013) 'Co-housing rodents with different coat colours as a simple, non-invasive means of individual identification: validating mixed-strain housing for C57BL/6 and DBA/2 mice', *PloS one,* 8(10), pp. e77541.
