# Supplementary material for: An exploration of the postural, location- and social contact- related sub-characteristics of inactive but awake behaviour as a depression-like indicator in mice
Source: Appl Anim Behav Sci. Author manuscript; Available in PMC 2025 Oct 22. (PMC7618284; doi:10.1016/j.applanim.2024.106431)
Supplement: Supplementary material 2 [file EMS207934-supplement-Supplementary_material_2.docx]

**An exploration of the postural, location- and social contact- related sub-characteristics of inactive but awake behaviour as a depression-like indicator in mice**

Anna C Trevarthen^*a^, Agustina Resasco^b^, Emily M Finnegan^a^, Elizabeth S Paul^a^, Michael T Mendl^a^, Carole Fureix^a^

^a^ University of Bristol, Bristol Veterinary School, Langford House, Langford BS40 5DU, United Kingdom

^b^ Biological Research Facility, The Francis Crick Institute, 1 Midland Rd, London NW1 1AT, United Kingdom

Authors’ email addresses: [anna.trevarthen@bristol.ac.uk](mailto:anna.trevarthen@bristol.ac.uk); [agustina](mailto:agustinaresasco@gmail.com).resasco@crick.ac.uk; emf4@hotmail.co.uk; [e.paul@bristol.ac.uk](mailto:e.paul@bristol.ac.uk); [mike.mendl@bristol.ac.uk](mailto:mike.mendl@bristol.ac.uk); [carole.fureix@bristol.ac.uk](mailto:carole.fureix@bristol.ac.uk).

* correspondence: Bristol Veterinary School, Dolberry Building, Langford House, Bristol, BS40 5DU, United Kingdom

**Supplementary material – refining the Inactive But Awake behaviour (‘IBA’) duration cut-off while scan sampling IBA**

*Methods*

Previous work has characterised IBA in mice as having a duration of at least 15s (Fureix *et al.*, 2016; Harper *et al.*, 2015). As part of experiment 1, we aimed to further refine our duration cut-off for IBA by documenting the length of each visible bout. After two afternoon sessions performing practice observations on the mice and developing an ethogram, an independent (from the scan sampling) experimenter (AR) began performing focal observations. Observations were carried out over four days per week during the middle four weeks of the experiment (the two weeks preceding and two weeks post environmental adjustment). All mice were observed at least once during each observation day following a pre-determined pseudorandom order, to ensure individuals were observed both during the morning and the afternoon across the course of each week. Each focal observation lasted minimum 3 minutes and the experimenter recorded whether the mouse was performing IBA at the beginning of the observation period. These bouts were then excluded from the analysis as the total bout duration was unknown. The duration of all visible IBA bouts were recorded, regardless of the length (*i.e.* bouts which were shorter than the previously defined 15s cut-off were also recorded). If the focal mouse was performing IBA at the end of the 3-minute observation period, the experimenter continued to observe until the mouse changed behaviour to record the total IBA bout duration.

To update our IBA duration cut-off formally, we performed a series of regression analyses in SPSS (version 24) using the frequency of bouts observed as the dependent variable and the bout duration as the independent variable. We iteratively removed the shortest bout duration (*i.e.* the first regression included all data, the second regression excluded bout durations of 1s etc.). We then compared the slope coefficients (by plotting beta values and confidence intervals from each regression model) to find the point at which the slope gradients were significantly different. We followed Cumming (2009) to identify when the overlap between the two sets of confidence intervals was less than 50% (*i.e.* the extreme bound of the confidence interval of one regression must be above (or below) the mid-point of the upper (or lower) confidence interval for the other regression to be classed as significantly different).

*Results*

Four hundred eighty-three IBA bouts were recorded overall (**Supplementary Material Figure 1**), of which a large proportion (n = 192 bouts) had a bout duration of 1 second, which we suspected were behavioural transitions rather than IBA bouts.


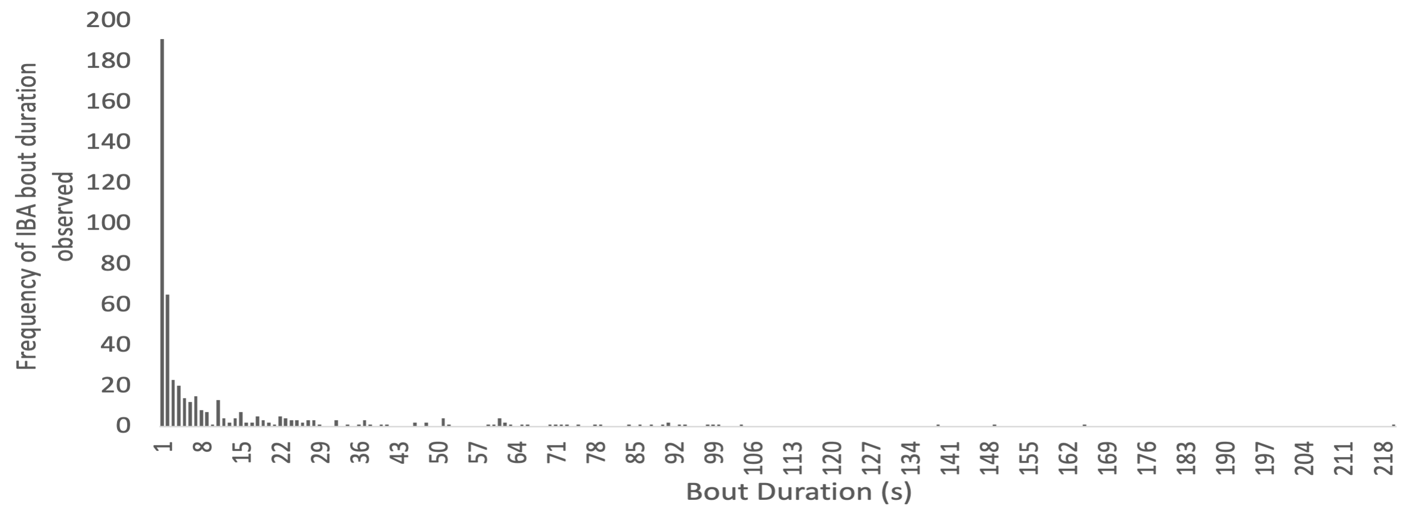


**Supplementary Material Figure 1:** Frequency distribution of IBA bouts observed during the four weeks of focal observations

When running the regression analyses, we found a significant overlap (>50%) between the confidence intervals of the regressions including and excluding 1s IBA bout durations (**Supplementary Material Figure 2a**). However, when both 1 and 2s IBA bouts were excluded from the regression, the overlap was less than 50% suggesting a significant difference between the slopes of the two regressions (**Supplementary Material Figure 2b**).

(a)

**(b)**

**Supplementary Material Figure 2:** The beta coefficient and 95% confidence intervals resulting from **a)** two regression analyses including and excluding 1s bout durations and **b)** two regression analyses including and excluding bout 1 and 2 s bout durations. The dashed line indicates the 50% overlap point between the confidence intervals. Following the procedure outlined in Cumming (2009), the values are significantly different when the confidence intervals from one regression analysis fall outside the 50% overlap mark.

Overlaps remain less than 50% for any further analyses (*i.e.* excluding 1, 2 and 3s IBA bouts, excluding 1, 2, 3 and 4s bouts etc) (**Supplementary Material Figure 3**). This enabled us to formally update our IBA cut-off to exclude 1 and 2 second immobility bout durations and include as IBA those with a duration of 3 seconds and above for further studies conducted both within (see experiment 2 this paper) and between laboratories (*e.g.* Nip *et al.*, 2019).

**Supplementary Material Figure 3:** The beta coefficient +/- 95% confidence intervals for the first 15 regression analyses performed.

*Supplementary material references*

Cumming, G. (2009) 'Inference by eye: Reading the overlap of independent confidence intervals', *Statistics in Medicine,* 28(2), pp. 205-220.

Fureix, C., Walker, M., Harper, L., Reynolds, K., Saldivia-Woo, A. and Mason, G. (2016) 'Stereotypic behaviour in standard non-enriched cages is an alternative to depression-like responses in C57BL/6 mice', *Behavioral Brain Research,* 305, pp. 186-190.

Harper, L., Choleris, E., Ervin, K., Fureix, C., Reynolds, K., Walker, M. and Mason, G. (2015) 'Stereotypic mice are aggressed by their cage-mates, and tend to be poor demonstrators in social learning tasks', *Animal Welfare,* 24, pp. 463-473.

Nip, E., Adcock, A., Nazal, B., Maclellan, A., Niel, L., Choleris, E., Levison, L. and Mason, G. (2019) 'Why are enriched mice nice? Investigating how environmental enrichment reduces agonism in female C57BL/6, DBA/2, and BALB/c mice', *Applied Animal Behaviour Science,* 217, pp. 73-82.
